# The Relationship between Hysterectomy and Depression: *Avicenna’s* Viewpoint

**DOI:** 10.31661/gmj.v8i0.1638

**Published:** 2019-12-31

**Authors:** Fatemeh Gholinia Navaee, Ali Aminian, Seyde Sedighe Yousefi

**Affiliations:** ^1^Department of Traditional Persian Medicine, Faculty of Medicine, Mazandaran University of Medical Sciences, Sari, Iran

**Keywords:** Hysterectomy, Depression, *Aviccena*, Modern Medicine

## Dear Editor,

Hysterectomy is the most common surgical procedure in non-pregnant women and the second common procedure, occurring post-cesarean section [1]. It is an effective solution for benign and malignant gynecological pathologies cause abnormal uterine bleeding like endometriosis, adenomyosis, leiomyoma, uterine polyps, and leiomyosarcoma [1]. In the United States, 600,000 women undergo hysterectomy every year [1], in which 75% of them are at the ages of 20-49 years old [2]. The usual complications of hysterectomy are physical and mental, including vaginal dryness, dyspareunia, changes in sexual activity such as decreased libido and also depression [3]. The World Health Organization (WHO), considers depression as one of the most commonly diagnosed diseases in the world, with the highest rates of ages between 25 to 44 years old [4]. The overall risk of sustained and chronic depression is more common in women [4]. On the other hand, recent studies in Modern medicine, have shown strong evidences of post-hysterectomy depression [5]. Persian medicine (PM), is one of the most important branches of complementary and alternative medicine, rooted in more than seven thousand years ago, studied by outstanding scientists especially those who lived in the golden age of Islam (9th to 13th century A.D) such as *Avicenna* (980-1037 A.D)[6]. It plays an important role in the prevention and treatment of diseases from the past to the present [6]. According to the holistic viewpoint of PM, the human body is made up of four main substances called “Humor” (*Khelt*), each of which has a unique temperament (*Mizaj*), and based on temperament, they play certain roles in the body [7]. As other PM philosophies, *Avicenna* believed that the balance of these four humors lead to health and imbalance of each one, in quality, quantity, or both causes various types of disease, classified as a category named “*Su-e-Mizaj*” (substantial dystemperament) [7, 8]. *Avicenna* emphasized that concurrent with normal menstruation cycle, some amount of waste materials are removed from the body, and if the menstruation cycle does not happen, these will accumulate and cause multiple complications in different organs, including the gastrointestinal tract, respiratory tract, uterus, liver, kidney, and especially central nervous system [8]. *Avicenna*, in his well-known book *Canon of Medicine*, (*Al-Qanoon- fi -Al-Tibb*), has been pointed out the neurological complications such as depression with the term “*Melancholia*” [8]. Cleansing the body from waste materials through urine, stool, sweating and menstruation (in women), accompanying with preserving essential substances, are the two important principles from the six essential principles of a healthy lifestyle in PM which known as “*Setteh-e-zarurieh*” [9]. According to this, if what is to be repelled remains, the natural body temperature affects the residual materials and rotten them, and by releasing these corrosive substances throughout the body, these can lead to serious harms. The nature of our body tries to preserve its balance by eliminating waste, and menstruation cycle, as a natural cleansing method, has a special role in women of reproductive age [9]. *Avicenna* believed that if normal menstruation cycle, named in PM as "Tams", as a natural way to clean up the body, is disrupted for any reason, the physician should use alternative purging interventions to prevent complications such as depression, and if depression occurred, prevent its progression [8]. Important *Avicenna’s* recommendations in this regard include the following: Lifestyle modification, especially exercise and daily physical activity, regulating the bedtime, having a proper diet, improving digestion especially by focusing on constipation, accurate counseling to solve psychological problems before and after hysterectomy and at last, using interventions [8]. The Venesection (*Fasd*), Wet cupping (*Hijamat*) and Leech therapy are various manual interventions known in PM as “*Amal-e-Yadavi*,” that have been used frequently [9]. As in *Avicenna’s* manuscripts, venesection is the most effective method to prevent the accumulation of waste materials in the body, following hysterectomy [8]. Although the onset of hysterectomy-induced depression based on the PM training is quite expected, this finding has not been conclusively proven yet. If this hypothesis is supported by future researches in Modern medicine, the recommendations of PM could be a good suggestion for treating or preventing post-hysterectomy depression.

## Acknowledgment

This article is based on ph.D. thesis of Fatemeh Gholinia Navaee (Thesis No: IR.MAZUMS.REC.1397.157) sponsored by Mazandaran University of Medical Sciences, Sari, Iran.

## Conflict of Interest

The authors declare that there is no conflict of interest.
